# Type 2 Diabetes Mellitus: Pathogenic Features and Experimental Models in Rodents

**DOI:** 10.32607/actanaturae.11751

**Published:** 2022

**Authors:** I. G. Gvazava, M. V. Karimova, A. V. Vasiliev, E. A. Vorotelyak

**Affiliations:** Institute of Developmental Biology, Russian Academy of Sciences, Moscow, 119334 Russia; Lomonosov Moscow State University, Faculty of Biology, Moscow, 119234 Russia

**Keywords:** type 2 diabetes mellitus, pathogenesis, insulin resistance, beta cells, experimental animals, streptozotocin

## Abstract

Type 2 diabetes mellitus (T2DM) is the most common endocrine disorder (90%) in the world; it has numerous clinical, immunological, and genetic differences from type 1 diabetes mellitus. The pathogenesis of T2DM is complex and not fully clear. To date, animal models remain the main tool by which to study the pathophysiology and therapy of T2DM. Rodents are considered the best choice among animal models, because they are characterized by a small size, short induction period, easy diabetes induction, and economic efficiency. This review summarizes data on experimental models of T2DM that are currently used, evaluates their advantages and disadvantages vis-a-vis research, and describes in detail the factors that should be taken into account when using these models. Selection of a suitable model for tackling a particular issue is not always trivial; it affects study results and their interpretation.

## INTRODUCTION

For many decades, diabetes mellitus (DM) has been one of the major health challenges in the world due to its associated increased morbidity, disability, and mortality. According to the International Diabetes Federation (IDF), there were 537 million people with DM in 2021; this number could reach 643 million by 2030, while the actual prevalence of DM is several times higher than what is on record [1]. DM is a chronic disease that develops either when the pancreas does not produce enough insulin (the hormone regulating blood sugar level) or when the body cannot effectively use the hormone it produces. A common consequence of uncontrolled DM is hyperglycemia, or elevated blood sugar, eventually leading to severe damage to numerous body systems, especially the nerves and blood vessels [2].

The presented review is a logical follow-up to our work that summarizes data on the pathogenesis of type 1 DM (T1DM) and considers the most commonly used experimental animal models as the most important tool in studying DM. The mechanisms of the most adequate and easily reproducible DM model, namely the streptozotocin-induced model of DM, were analyzed and discussed in [3]. Type 2 diabetes mellitus (T2DM) is the most common endocrine disease; it is diagnosed in more than 90% of all diabetic patients. T2DM symptoms may be similar to those of T1DM, although they are often less severe [2]. This review will focus on the pathogenic mechanisms of T2DM onset and progression, as well as modeling of various disease stages, in rodents for further use in the search for new therapeutic agents and treatments for T2DM.

T1DM and T2DM have numerous clinical, immunological, and genetic differences. T2DM (non-insulin-dependent, or adult-onset diabetes) develops as a result of inefficient use of insulin by the body. The disease is often diagnosed several years after its onset, when complications develop. Until recently, T2DM was observed only in adults; now it is increasingly prevalent in children, since childhood obesity, which is associated with DM, has become an epidemic [2]. For a long time, there has been an erroneous belief that T2DM is a mild form of the disease, and that it might develop without complications. However, to date, researchers firmly believe that the condition is a severe chronic progressive disease, with more than 50% of patients having late complications by the time of the diagnosis. The high prevalence of T2DM among some ethnic groups and patients’ relatives points to the existence of genetic factors that are associated with the disease. In recent years, several genetic polymorphisms associated with DM have been identified; however, no single gene responsible for the most common form of DM, namely non-insulin-dependent DM, has been identified. There are considered to be two subtypes of T2DM: with mutations in individual genes (10–15%) and damage to a set of genes (85–90%) responsible for insulin binding to a cell receptor, internalization of the hormone–receptor complex, autophosphorylation of β receptors, and phosphorylation of other membrane protein components. An example of multiple damage is the insulin resistance (IR) of cells caused by multiple mutations in the insulin receptor gene. Up to 30 different mutations have been identified in this gene [4, 5, 6, 7, 8, 9].

T2DM is a multifactorial disease characterized by a large heterogeneity of metabolic defects, the most common of which are insufficient insulin production, IR, and incretin system defects. It is important to understand the multifactorial nature of T2DM, which is determined by the combinatorial effect of genes and the environment. Therefore, there is no simple genetic and epidemiological model that explains the disease inheritance. Hence the need to establish how much of the disease is determined by genes and what is the contribution of environmental factors, the combination of which regulates the threshold/level of tolerance to DM development [6, 7].

Despite the availability of modern treatment strategies, T2DM remains a pressing issue for the healthcare system worldwide. This is mainly due to an increase in the disease incidence associated with factors such as aging and the growth of obesity in the population. The risk of T2DM grows higher with age. Excess weight and obesity contribute to the development of IR and hyperglycemia. The progressive course of T2DM is an indication that lifestyle changes are not enough to achieve and maintain glycemic control; most T2DM patients require drug treatment [4, 5].

T2DM is relatively easy to diagnose when symptoms are present. However, according to the United Kingdom Prospective Diabetes Study (UKPDS), T2DM may remain undiagnosed for many years. It takes from three to six years for T2DM to be diagnosed from the moment of the disease onset. Therefore, early T2DM diagnosis remains relevant, especially in individuals at high risk of developing the disease. More than half of patients already have several complications by the time of their diagnosis. Severe retinopathy is found in 20–40% of patients. The development of diabetic complications, such as retinopathy, nephropathy, and neuropathy, is due to long-term hyperglycemia. This fact points to the necessity and importance of monitoring blood sugar levels [2].

The pathogenesis of T2DM is complex and not yet fully understood. To date, IR, impaired insulin secretion, increased glucose production by the liver, as well as hereditary predisposition, lifestyle, and nutritional habits leading to obesity, are considered to be the key elements of T2DM pathogenesis. Hyperglycemia develops when insulin secretion is no longer able to compensate for IR. Although IR is characteristic of T2DM patients and at-risk individuals, there is evidence of β-cell dysfunction and related disorders of insulin secretion, including the first phase of secretion in response to intravenous glucose infusion, impaired physiological pulsatile secretion of insulin, increased secretion of proinsulin, which indicates impaired insulin processing, and the accumulation of amyloid in pancreatic islets (which is normally secreted together with insulin). The decrease in the β-cell mass and function is of fundamental importance in T2DM pathogenesis. The loss of β-cell mass is poorly understood, although increased β-cell loss is considered to contribute to IR. The proposed mechanisms responsible for the loss of β-cells in T2DM include amyloid formation and endoplasmic reticulum stress; however, their relative contribution remains unknown. The pathology of Langerhans islets in T2DM, which is extremely heterogeneous, is worth attention. For example, many Langerhans islets look completely normal, some islets contain large amyloid deposits, while others do not. The differences in the age of β-cells is believed to be one of the factors underlying their heterogeneity [10, 11]. Amyloid formation in the Langerhans islets has a toxic effect on hormone-producing islet cells, leading to pancreatic damage. As a result, hyperproduction of hormones in T2DM is replaced by their deficiency [12 , 13, 14]. Hyperglycemia alone can affect insulin secretion, since high glucose levels cause cell desensitization and/or dysfunction (glucose toxicity). These changes in the presence of IR usually develop over many years [10, 11, 15, 16]. An important condition for IR to develop in T2DM is obesity and weight gain. Obesity can be determined by genetic factors. However, di etary preferences, exercise intensity, and lifestyle in general also play an important role. The body cannot suppress lipolysis in adipose tissue, so free fatty acids are released from it, and their increased plasma levels can impair insulin-stimulated glucose transport and muscle glycogen synthase activity. Adipose tissue also functions as an endocrine organ, secreting many factors (adipocytokines) to the blood that positively (adiponectin) or negatively (tumor necrosis factor-α (TNF-α), interleukin 6, leptin, and resistin) affect glucose metabolism. Intrauterine growth retardation and low birth weight are also associated with IR development in older age, which may indicate the adverse prenatal effect of environmental factors on glucose metabolism. Currently, IR is mostly associated with impaired insulin action at the post-receptor level; in particular, with a significant decrease in the membrane levels of specific glucose transporters (GLUT-4, GLUT-2, and GLUT-1) [5 , 6, 7, 17, 18].

According to modern concepts of the cellular and molecular mechanisms of T2DM, IR – or a decreased biological response of cells to one or several effects of normal blood levels of insulin – is the first element in the disease’s pathogenesis. IR leads to the inability of insulin-dependent (muscle and adipose) tissues to absorb glucose from plasma and a disruption of glycogen (glucose polymer) synthesis in the liver. The fine mechanisms of IR development in T2DM are not yet fully understood. Although the exact cause of IR has not been elucidated, a number of underlying mechanisms have been suggested: oxidative stress, inflammation, insulin receptor mutations, endoplasmic reticulum stress, and mitochondrial dysfunction [19, 20, 21, 22, 23, 24]. IR is known to affect the activity of the enzymes of glycolysis and gluconeogenesis, glycogen synthesis and glycogenolysis, β-oxidation of fatty acids, and lipogenesis. Insulin inhibits the mobilization of fats and intake of free fatty acids circulating in the blood by cells, potentiates protein synthesis in almost all tissues, primarily skeletal muscles, myocardium, and liver, and also affects the capture and transport of amino acids, which comprise all proteins, and major ions. Normally, a two-chain insulin molecule binds to a specific receptor located on the cell membrane carrying a tyrosine kinase fragment with enzymatic activity, which triggers tyrosine autophosphorylation, followed by the activation of the proteins involved in secondary signal transduction (insulin receptor substrate-1 (IRS1), Shc-1, SIRP-α, Gab-1, Cbl-b, etc.). IRS1 proteins activate phosphatidylinositol 3-kinase, which, in turn, triggers the action of protein kinases B. Protein kinases B and C initiate a cascade of enzymes that regulate carbohydrate and fat metabolism and lead to the incorporation of glucose transporters (GLUT-4) into the membranes of insulin-dependent cells (adipocytes and myocytes). This is how glucose molecules are transported from the blood plasma into cells [18, 19, 24, 25]. The mechanisms of nitric oxide synthesis in vascular endothelial cells in muscle tissue, intensive uptake of amino acids and synthesis of cellular proteins, as well as inhibition of apoptotic processes are triggered, together with the activation of glucose intake. Another group of proteins responsible for secondary signal transduction from the insulin receptor (Shc-, Sos-, Ras-, Raf-, and Map-) regulates the mechanisms of mitosis and cell proliferation and activates the synthesis of inflammatory mediators. A detailed study of the pathway of insulin action on intracellular processes allows us to imagine the versatility of the potent factors involved in IR development. Molecular causes behind the loss of the ability to transmit a signal may be the suppression of the activity of IRS1 tyrosine kinase and phosphatidylinositol 3-kinase due to various mutations in the gene encoding the insulin receptor. Impaired glucose entry into the cell can be caused by either a decrease in the efficiency of protein kinases B and C or structural deficiency of the transmembrane glucose transporter (GLUT-4). All of the abovementioned mechanisms of IR development can be congenital, genetically determined; they are described for a series of syndromes. The biological effects of insulin are much more often impaired during life due to the effect of additional factors. A decrease in the tyrosine kinase activity of the insulin receptor is currently considered the key mechanism undergirding the development of acquired IR. Membrane glycoprotein PC-1, which is produced in excess by myocytes and adipocytes, is a known factor involved in the disruption of the tyrosine kinase element in intracellular signal transmission. Inhibitors of tyrosine kinase effects, namely protein kinase C and TNF-α, are also synthesized by adipocytes in large amounts [5, 18, 19].

Another factor involved in IR development is a decrease in the activity of phosphatidylinositol 3-kinase due to either imbalance of its subunits caused by certain hormones (glucocorticoids and sex steroids) or excessive intake of free fatty acids and triglycerides by cells, leading to diacylglycerol accumulation. Adipose tissue plays an important role in the energy homeostasis of the whole organism and regulation of metabolic functions. It serves as a deposit of excess energy, in the form of triglycerides, in adipocytes and regulates lipid mobilization during fasting by releasing free fatty acids [24, 26, 27]. With the discovery of adipocyte-derived factors such as leptin, adiponectin, and resistin, adipose tissue is recognized as a complex endocrine organ. Adipose tissue can attach to many organs (liver, pancreas, muscles, and the brain) through adipokine signaling and modulate systemic metabolism [27, 28, 29, 30, 31]. Thus, adipose tissue dysfunction plays an important role in the pathogenesis of such metabolic disorders as obesity, IR, and DM [32].

In addition to the described general mechanisms of impaired insulin action, the biologically active substances produced by adipocytes, which have a profound effect on systemic metabolism, play the most important role in IR development in the case of excessive growth of adipose tissue. Adipocyte-derived metabolites (adipocytokines) can affect various biochemical processes in many organs and tissues. Currently, more than 100 chemical compounds of similar origin are known, many of which are directly or indirectly associated with IR [27, 33, 34].

The peptide hormone leptin (Lep), one of the first identified adipocytokines, is encoded by the ob gene (obesity gene) [35, 28]. In addition to adipocytes, many tissues and organs (liver, muscles, ovaries, etc.) also produce Lep, which indicates the diversity of its biological effects. Lep occupies a central place in the regulation of energy homeostasis and body weight. The most studied mechanism of the hormone’s action is the stimulation of the satiety center located in the hypothalamus. Normally, Lep in mammals exerts anorexigenic, catabolic, lipolytic, and hypoglycemic effects, thus triggering a negative feedback mechanism. In obesity, the action of Lep is impaired due to the inhibition of its normal transport through the blood– brain barrier or binding to the receptor form circulating in the blood [36, 37].

The feeling of hunger decreases or completely disappears with an increase in Lep blood levels. However, a log-term and persistent increase in the hormone level causes leptin resistance: the resistance of target cells in the hypothalamus to its effects. Lep resistance leads to excess intake of triglycerides and free fatty acids by the cells of insulin-dependent tissues, leading to IR [38].

Adiponectin is produced exclusively by adipocytes and plays an important role in the regulation of lipid and carbohydrate (glucose) metabolism, increasing the sensitivity of adipose and muscle tissues to insulin. The intracellular effects of adiponectin are achieved through the activation of AMP kinase and phosphatidylinositol 3-kinase, which regulate the oxidation of free fatty acids. Adiponectin decreases the production of inflammatory mediators (interleukin 6, interleukin 8, TNF-α, etc.) and the metalloproteases inhibiting the function of insulin receptor tyrosine kinase (IRS1) [39, 40, 41]. A decrease in the adiponectin level with excessive development of adipose tissue via the feedback mechanism (decrease in hormone production upon reaching the required level of its effect: accumulation of energy deposit of cells) is one of the factors behind IR development [42, 43].

The sensitivity of adipose and muscle tissues to insulin is also affected by an adipocytokine with a studied mechanism of action: resistin. Angiotensinogen and a number of other hormone-like substances produced by adipose tissue cells have a similar effect [44, 45].

Loss of tissue sensitivity to insulin leads to compensatory hyperproduction of the hormone by pancreatic β-cells. An increase in the plasma levels of insulin for some time makes it possible to overcome the IR barrier, while maintaining the required level of glucose intake by the cells. However, the storage capacity of the insular apparatus of the pancreas gradually becomes exhausted and leads to decompensation: namely, DM [46].

Much attention is paid to the development of innovative technologies to combat DM. Despite the tremendous progress achieved in molecular genetic research in the field of T2DM [47, 48, 49, 50, 52, 52], measures for its prevention and treatment have not been developed at the proper level yet.

It is known that success in theoretical research and the development of methods for disease prevention and treatment cannot be achieved without disease modeling in experimental animals and depends on a correct choice of the model animal. Valuable data that can help understand the mechanism of the antidiabetic action of various agents for their subsequent targeted application can be obtained only with the use of experimental models that are closest to the disease etiology and pathogenesis. An objective analysis of the advantages and disadvantages of each model, depending on the established goal, will go a long way in helping avoid erroneous results [3].

T2DM is considered to be a complex, genetically heterogeneous human disease whose pathogenesis is determined by both inheritance and environmental factors in general. T2DM is studied by disease modeling in mice and rats. Rodents are considered the best choice among animal models, because they are relatively inexpensive to maintain; they reproduce rapidly, allowing genetic effects to be studied in several generations within a reasonable period of time; and, very importantly, because the rodent genome shares a more than 90% similarity with the human genome [53]. Rats are more preferable than mice, since it is easier to perform surgery on rats owing to their larger size; in addition, they are more resistant to various diseases.

In this study, we continue to analyze the existing experimental models in order to identify the most suitable and available model for studying T2DM. The pathogenesis and laboratory models of T1DM are described in our previous work [3].

Rodent models of T2DM fall into two main categories: genetic (spontaneously induced) and non-genetic (experimentally induced) models. Non-genetic models are known to be more common than the genetic ones due to their lower cost; greater availability; easier DM induction; and, of course, simpler composition [3].

Because T2DM is characterized by IR and the inability of β-cells to adequately compensate for it, animal models of T2DM typically include modeling of IR and/or β-cell deficiency. Many animal models are obese, similar to the human condition in which obesity is strongly associated with T2DM development. Obesity can result from naturally occurring mutations, genetic manipulations, and consumption of high-fat foods.

## GENETIC MODELS WITH OBESITY

**Monogenic models**

The following rodents are the most widely used as monogenic models of obesity to test new methods for treating T2DM: Zucker rats with diabetes and obesity (Zucker diabetic fatty, ZDF), Lep ob/ob and Lepr db/db mice with Lep deficiency [36, 37]. Impaired Lep reception is observed in these models in obesity. A homozygous mutation in LEPR makes the corresponding receptor non-functional. On the one hand, these animals lack the effect of fat reserves on the amount of food consumed, which leads to rapid development of obesity even in a standard, balanced diet. On the other hand, the disruption of Lep reception and internalization by cells impedes its clearance, leading to a sharp increase in the blood level of the hormone and development of immunotropic effects that are normally absent and caused by partial homology between the structures of Lep and a number of cytokines and chemokines [54, 55]. Expression of a large number of the genes involved in the various metabolic pathways that determine changes in body homeostasis is altered in the organs and tissues of these animals. A metabolic imbalance emerges in the body. Since Lep induces satiety, the lack of the functional hormone causes hyperphagia and subsequent obesity in these animals [56, 57, 58]. These changes are largely consistent with those in patients with alimentary obesity.

Lep ob/ob mice derive from animals with a spontaneous mutation found in an outbred colony at Jackson’s laboratory in 1949. Mice with this phenotype were crossed with C57BL/6 mice, but it was not until 1994 that the mutant protein was identified as Lep [59]. These mice gain weight and develop hyperinsulinemia by two weeks of age. By week 4, hyperglycemia becomes apparent and the blood level of glucose continues to grow, peaking at 3–5 months of age and then decreasing as the mouse matures. Animals also experience hyperlipidemia, impaired thermoregulation, and decreased physical activity. Pancreatic hypertrophy is observed. Despite impaired insulin clearance, islets maintain secretion, which does not make this model fully representative of T2DM in humans. However, C57Bl/KS mice develop much more severe diabetes, with islet atrophy and early mortality. In addition, these mice are sterile [60, 61].

Lepr db/db mice were obtained at the Jackson’s laboratory as a result of an autosomal recessive mutation in the Lep receptor. These mice develop hyperinsulinemia at two weeks of age, obesity and hyperphagia at week 3–4 of age, and hyperglycemia at week 4–8. The most commonly used strain is C57BLKS/J; these mice develop ketosis at the age of several months and have a relatively short lifespan [62, 63].

Zucker rats offer a classical model to study obesity, T2DM, hypertension, and cardiac dysfunction. These rats were named after Columbia University pathologists Louis and Theodore Zucker, who discovered a gene responsible for obesity in rats in 1961. Zuckers crossed Merck M and Sherman mice and revealed a spontaneous recessive mutation fa (fatty) in Lepr, the gene encoding the receptor to the satiety hormone Lep. The mutant Lep receptor causes obesity in these rats at week four [64]; the animals also develop hyperinsulinemia, hyperlipidemia, hypertension, and impaired glucose tolerance [63]. Mutation in these rats led to the emergence of a rat substrain with a diabetogenic phenotype: inbred ZDF rats, diabetic obese Zucker rats. These rats are less obese than Zucker rats but have more pronounced IR. It is impossible to compensate for IR in these animals due to the increased apoptosis in their β-cells [65]. Hyperinsulinemia is observed at about eight weeks of age, followed by a decrease in the insulin levels [66]. Diabetes usually develops around week 8–10 in males; females do not develop overt diabetes. These rats also show signs of diabetic complications [63].

**Polygenic models**

In contrast to the monogenic models described above, polygenic models of obesity can provide a more accurate model of the disease in humans. Numerous polygenic murine models of obesity, glucose intolerance, and diabetes are known, which makes it possible to perform a detailed study of different genotypes and their susceptibility. However, polygenic models (unlike monogenic ones) lack wild-type controls but demonstrate sexual dimorphism, with a preference for males [67]. Polygenic models, namely KK and KK AY mice, OLETF rats, NZO mice, etc., are characterized by obesity-induced hyperglycemia, severe hyperinsulinemia, IR in both muscle and adipose tissue, and pronounced changes in the pancreatic islets: from hypertrophy and degranulation to fibrosis and their replacement by the connective tissue [67, 68, 69, 70]. A number of works focused on the elimination of T2DM symptoms, analysis of the relationship between obesity and glucose homeostasis, as well as diabetic complications, have been done using polygenic models [71 , 72, 73, 74, 75, 76, 77, 78, 79, 80, 81, 82, 83, 84, 85, 86].

**Induced obesity models**

A high-fat diet leads to obesity. The model for feeding C57BL/ mice a high-fat diet was first presented in 1988 [87]. It has been shown that mice fed a high-fat diet (about 60% of fats) can weigh more than a control group fed a standard diet after a week. Using this diet for several weeks causes a more pronounced weight gain, associated with IR, while the lack of compensation of β-cells leads to impaired glucose tolerance [88]. The obesity in this model is considered to be caused environmentally, rather than determined genetically; hence, it is more similar to the disease in humans compared to genetic models of obesity-induced diabetes. It has been shown that, in transgenic and knockout models, which may not show an overt diabetic phenotype in normal conditions, a high-fat diet stimulates β-cells and the gene starts to play an important role. The susceptibility to diet-induced metabolic changes depends on the mouse’s strain. Thus, the effects may be left unnoticed in case of using a more resistant strain [89, 90, 91, 92, 93, 94, 95]. For example, inbred C57BL/6 mice are characterized by heterogeneity in response to a high-fat diet. However, differential responses to a high-fat diet are not always in place even when using genetically homogenous rats and mice [96].

Rodents defined as **useful models **are used to study T2DM. They include the desert gerbil (Psammomys obesus; first discovered in 1960) and the recently described Nile grass rat (Arvicanthis niloticus) [97]. Most of these animals when kept in captivity with a normal diet for a year spontaneously develop diabetes that progresses from mild hyperglycemia with hyperinsulinemia to severe hyperglycemia with hypoinsulinemia and ketoacidosis. Progression from one stage to another can be prevented by restricting food intake. However, recovery from the final hyperglycemic/insulinopenic stage is impossible. Although these rodents are not hyperphagic, constant availability of a high-calorie diet results in obesity, dyslipidemia, hyperglycemia, as well as other signs of diabetes and metabolic syndromes, such as decreased β-cell mass, atherosclerosis, and hepatic steatosis, in them. Because of poor adaptation to overnutrition, P. obesus may be an ideal model for the thrifty gene effect, due to which the animal often develops IR and the metabolic syndrome after a rapid switch from food deficiency to excess. These animals are a valuable spontaneous model for research aimed at preventing diet-induced diabetes and represent a novel system of the interactions between genes and diet that affect energy use. This model will allow for a better understanding of the approaches to prevent and treat T2DM and the metabolic syndrome [97, 98, 99, 100, 101].

**Models without obesity**

However, not all T2DM patients are obese; thus, T2DM modeling in non-obese animals with β-cell dysfunction is indeed necessary [102]. Goto-Kakizaki (GK) rats are the most common non-obese models of T2DM [103]. This model was obtained by multiple crossing of Wistar rats, which are characterized by the worst glucose tolerance. It is assumed that IR is not the main initiator of hyperglycemia in this model, and impaired glucose metabolism is considered a consequence of reduced β-cell mass [104] and/or their aberrant function [105]. The effect of the morphology of pancreatic Largenhans islets on their metabolism varies in different rat colonies. For example, in some of them (Stockholm and Dallas colonies), the volume and density of β-cells are similar to those of the control; apparently, hyperglycemia is caused by the defects in insulin secretion, while a decrease in the β-cell mass is observed in the Paris colony of GK rats [105]. GK is one of the best characterized animal models of spontaneous T2DM; it is suitable for studying crucial aspects of the disease. The defective β-cell mass and function in the GK model are believed to be a reflection of complex interactions between multiple pathogenic factors. These factors include several independent loci containing the genes responsible for some diabetic features (except for a decrease in β-cell mass), gestational metabolic disorder inducing epigenetic programming of the pancreas (decreased neogenesis and/or β-cell proliferation) that is transferred to the next generation, loss of β-cell differentiation due to chronic exposure to hyperglycemia/hyperlipidemia, inflammatory mediators, oxidative stress, and impaired islet microarchitecture [101]. GK rats have been used to study both β-cell dysfunction in T2DM [106, 107, 108, 109] and diabetic complications [110, 111].

hIAPP mice. Human T2DM is characterized by amyloid formation in the islet tissue derived from islet amyloid polypeptide (IAPP) [10, 112, 113]. In addition to humans and macaques, pancreatic islets in cats also produce amyloid, which makes this animal a good model to study islet amyloidosis. This aspect of the disease is not usually modeled in rodents, since rodent IAPP is not amyloidogenic [11, 12, 114, 115]. However, transgenic mice expressing human IAPP (hIAPP) under the insulin promoter have been developed; these mice can produce amyloid in their islets. Using a large number of hIAPP models, it has been shown that hIAPP overexpression increases β-cell toxicity [116]. In addition, replicating β-cells are more susceptible to hIAPP toxicity; therefore, this model limits the adaptation of β-cells to increased insulin requirements [117].

Knockout and transgenic mice are also used to create specific T2DM models. These models have become a powerful tool in elucidating the role of specific genes in the glucose metabolism and disease pathogenesis [63, 118]. The use of knockout and transgenic mice made it possible to identify the transcription factors involved in the pancreas development and insulin signaling pathways. Tissue-specific knockouts turned out to be particularly useful in studying insulin signaling, since mice with global knockout of the insulin receptor are not viable [119, 120, 121, 122, 123].

Although T2DM is the most common form of DM, model development is more difficult in the case of T2DM, compared to T1DM. Genetic models such as obese diabetic Zucker rats and db/db mice are perhaps the closest to the human disease. However, the use of these models is limited because they have some crucial differences, do not accurately reflect T2DM in humans [124], and are also expensive.

## STREPTOZOTOCIN MODELS OF T2DM

Streptozotocin models of T2DM (STZ T2DM) are the most commonly used animal models of T2DM. Two potentially useful STZ T2DM models have been developed. The model with simultaneous administration of nicotinamide to rats for partial protection of β-cells against the effects of STZ [125] is based on the fact that nicotinamide prevents the diabetogenic effect of STZ [126, 127]. This combination creates a model of insulin-deficient – but not insulin-resistant – T2DM, characterized by stable, moderate hyperglycemia associated with an approximately 60% loss of β-cell function [125, 128]. The use of this protocol results in moderate, non-fasting hyperglycemia in 75–80% of the animals, while the other animals either develop severe hyperglycemia after 2–3 weeks or remain normoglycemic but with glucose intolerance. The same protocol can be used in mice. It should be taken into account that the STZ dose and the time between administration of nicotinamide and STZ are of crucial importance. For example, if the STZ dose is too high or the time period between nicotinamide and STZ administration is too long, then a more severe insulin deficiency will be observed [129].

Since most T2DM patients have a combination of impaired insulin secretion and IR, another model has been developed to more closely mimic the human condition. To develop IR, animals were kept on a high-fat diet, followed by administration of moderate doses of STZ to cause β-cell dysfunction [130]. This resulted in hyperglycemia associated with hyperinsulinemia and IR [131]. The recommended diet provides 60% of calories from fat; a commercial, balanced diet should be used instead of the standard diet supplemented with fat [132]. The use of a high-fat diet to induce IR, followed by low to moderate doses of STZ to develop mild to moderate insulin deficiency, may currently be the most useful T2DM model. Animals kept on a high-fat diet are generally considered to be the best model for characterizing many complications associated with human DM [133].

An STZ dose should induce stable hyperglycemia in rats fed a high-fat diet for at least 130 days. If the STZ dose is too high, then the model represents T1DM and the mortality of the rats increases [134]. The use of two lower doses of STZ (30 mg/kg, intraperitoneally) administered at weekly intervals causes diabetes in 85% of the animals, with an average fasting blood glucose level of ~14 mmol/L (~252 mg/dL) [134]. Other researchers recommend using an STZ dose of 30 mg/kg intraperitoneally as the optimal dose for 12-week-old Sprague-Dawley rats fed a high-fat diet for eight weeks [135].

When STZ enters the bloodstream, it is delivered to pancreatic β-cells by glucose transporter protein 2 (GLUT-2). Inside the β-cells, STZ interrupts a number of important cellular processes and, if there is enough damage, it all culminates in DNA damage and cell death [3, 136, 137]. The final outcome of STZ administration is a decrease in the functional mass of β-cells, which manifests itself in insulin deficiency and further inability to metabolize glucose [137]. The combination of insulin deficiency with a high-fat diet, which requires elevated insulin levels to account for cellular IR [138, 139], leads to the glucose intolerance [140] characteristic of human T2DM. Prolonged mild β-cell damage results in more sustained and consistent fine effects than a single high dose. For example, administration of STZ using osmotic mini-pumps, in contrast to intraperitoneal and intravenous administration, provides significantly greater control over the resulting level of hyperglycemia while preserving the obesity phenotype [141]. The authors concluded that the observed overall increase in effectiveness is most likely due to the long-term effect on β-cells. In addition, the dose-dependent effect of STZ is due to a reciprocal reduction in the insulin secretory capacity and morphological changes in the pancreas. This model is considered capable of reproducing different stages of T2DM, which are defined by the dose-dependent effect of STZ on glucose intolerance. This method requires fewer animals to observe significant effects than the previously used methods [142, 143], when the animals either do not respond to STZ or die depending on the drug dose [141].

As noted earlier, despite the wide variety of animal models of DM described to date, preference is given to STZ-induced diabetes. The mechanism of STZ action, doses and ways of its administration, as well as species and gender differences in sensitivity to STZ are described in detail in the first part of our study [3]. The advantage of STZ-induced diabetes is the relative ease of reproduction, high selectivity, and the possibility of inducing DM of varying severity and duration, which makes it possible to simulate not only gradually developing β-cell dysfunction, but also impaired glucose tolerance and the disorders associated with it [3].

Thus, it is important to emphasize that the long course of T2DM in humans makes it difficult to model the disease and that additional animal models and techniques are required. It is important to develop animal models that accurately reproduce T2DM pathogenesis in humans, since this will allow to identify preventive and therapeutic strategies against T2DM and the complications associated with it. When studying T2DM, it is important to consider the mechanisms underlying hyperglycemia and their relevance to the study. These mechanisms may include IR and/or β-cell deficiency. Indeed, the conclusion on whether drug intervention can reduce symptoms in any given model may hinge on whether β-cells have failed. Models also vary in their physiological significance, with some being more reminiscent of disease progression than others. Models such as pancreatic regeneration are quite extreme, and it remains to be established whether the mechanisms of β-cell expansion in these models may play a role in DM development in humans.

The choice of the model depends on the study objective. Animal models that are useful for evaluating potential antidiabetic agents and diabetic complications have limited construct validity and are therefore less useful as tools to determine disease etiology [144].

Using rats and mice to model diabetes has clear advantages over other species; these advantages include animal size, short induction period, easy DM induction, and economic efficiency [145]. Mice have made a huge contribution to the understanding of human biology as an experimental animal. Mouse models are widely used to study human diseases because of the genetic homology between them [107]. As for DM, mouse models are invaluable for studying obesity and T2DM, determining the role of inflammation, IR, and potential treatment strategies [146 , 147, 148]. Rats are often used as a model to study the metabolic profile and pathologies associated with different T2DM stages [149]. Rat as an experimental model of human diseases has great advantages over mice and other rodents [150]. It is easier to study the physiology and accumulate information using rodents [142]. However, in order to gain insight into the diverse manifestations of DM in patients, it is highly advisable to use different models. More than one rodent species or strain should be studied, and the sex of the animal should also be considered, since many of the models described above, for example Zucker and OLETF rats and NZO mice, as well as many knockout and transgenic models of DM, are characterized by sexual dimorphism, which is not observed in humans [151]. It has been suggested that this is due to the action of sex hormones in some cases [152], although the exact mechanism of sexual dimorphism has not been elucidated. Indeed, the effects of sex hormones may vary in different mouse models; for example, gonadectomy in males prevents DM in some models, while being ineffective or even increasing disease incidence in others [151]. Sexual dimorphism may also include differences in mitochondria and stress responses [151]. When using knockout and transgenic mice, the presence of the hypothalamic syndrome and its effect on the phenotype should be excluded and appropriate controls are needed.

Experimental models are widely used to study drugs and the mechanisms underlying metabolic disorders. Since the prevalence and complications associated with DM continue to increase worldwide, DM models play a key role in the study of the disease’s pathogenesis and its complications in humans such as retinopathy, nephropathy, cardiomyopathy, and neuropathy. Despite all the advantages offered by these animals in the creation of new drugs, they come with individual restrictions that would limit the development of new agents and therapeutic interventions. Obese and non-obese animals with hyperglycemia, IR, and β-cell resistance are commonly used to study T2DM. Since experimental models differ in their physiological purposes and are used to study various complications of T2DM in humans, selection of a model for a particular study should be considered with great caution. In addition to the models used to elucidate the mechanisms underlying DM, various animal models are currently utilized to develop and validate new treatment strategies, most of which allow one to study some specific aspects of DM, while being of little use in other studies. All models have their pros and cons, and choosing the right one for a particular case is not always easy, since it affects the study results and their interpretation. When choosing a DM model, it is highly advisable to use a variety of different models to represent the diversity observed in diabetic patients. The number of available models is constantly on the rise, and it is important to consider their potential role in various aspects of the DM study.

Thus, despite the variety of biological models, the issue of a precise correspondence between the majority of experimental models and processes occurring in the human body remains unresolved. It is important that the results obtained during experimental modeling using laboratory animals represent a body of evidence that, with a certain degree of probability, can be extrapolated to humans.

Nevertheless, it is necessary to emphasize that, although the question of the extent to which the results extracted from biological models can be extrapolated to the human body is both the most important and the most difficult, experimental models remain our main tool for studying the pathophysiology and possible approaches to the treatment of DM [153].

## Abbreviations

T2DM: type 2 diabetes mellitus
DM: diabetes mellitus
T1DM: type 1 diabetes mellitus
β-cells: beta cells
STZ: streptozotocin
IR: insulin resistance
Lep: leptin
IAPP: islet amyloid polypeptide

## References

[1] The IDF Diabetes Atlas 10th Edition..

[2] (2016). Bulletin of the World Health Organization No. 312, April 2016..

[3] Gvazava I.G., Petrakova O.S., Rogovaya O.S., Borisov M.A., Terskih V.V., Vorotelyak E.A., Vasiliev A.V. (2018). Acta Naturae..

[4] Dedov I.I., Shestakova M.V., Galstyan G.R. (2016). Diabetes Mellitus..

[5] Dedov I.I., Tkachuk B.A., Gusev N.B., Shirinsky V.P., Vorotnikov A.V., Kocheruga T.N., Mayorov A. U., Shestakova M.V. (2018). Diabetes Mellitus..

[6] Balabolkin M.I. (2004). Medical Department..

[7] Zheng Y., Ley S.H., Hu F.B. (2018). Nat. Rev. Endocrinol..

[8] Permutt M.A., Wasson J., Cox N. (2005). J. Clin. Invest..

[9] De Rosa M.C., Glover H.J., Stratigopoulos G., LeDuc C.A., Su Q., Shen Y., Sleeman M.W., Chung W.K., Leibel R.L., Altarejos J.Y. (2021). JCI Insight..

[10] Weir G.C., Bonner-Weir S. (2013). Ann. N.Y. Acad. Sci..

[11] Aguayo-Mazzucato C., van Haaren M., Mruk M., Lee Jr.T., Crawford C., Hollister-Lock J., Sullivan B.A., Johnson J.W., Ebrahimi A., Dreyfuss J.M., Deursen J.V., Weir G.C., Bonner-Weir S. (2017). Cell Metab..

[12] Gudkova A.Y., Antimonova O.I., Shavlovsky M.M. (2019). Med. Acad. J..

[13] Sevcuka A., White K., Terry C. (2022). Life (Basel)..

[14] Bhowmick D.C., Singh S., Trikha S., Jeremic A.M. (2018). Handb. Exp. Pharmacol..

[15] Mukherjee N., Lin L., Contreras C.J., Templin A.T. (2021). Metabolites..

[16] Hu F., Qiu X., Bu S. (2020). Arch. Physiol. Biochem..

[17] Tokarz V. L., MacDonald P.E., Klip A. (2018). J. Cell. Biol..

[18] Thurmond D.C., Pessin J.E. (2001). Mol. Membr. Biol..

[19] Yaribeygi H., Farrokhi F.R., Butler A.E., Sahebkar A. (2019). J. Cell. Physiol..

[20] Bitar M.S., Al-Saleh E., Al-Mulla F. (2005). Life Sci..

[21] Sarparanta J., García-Macia M., Singh R. (2017). Curr. Diabetes Rev..

[22] Latouche C., Natoli A., Reddy-Luthmoodoo M., Heywood S.E., Armitage J.A., Kingwell B.A. (2016). PLoS One..

[23] Hasnain S.Z., Prins J.B., McGuckin M.A. (2016). J. Mol. Endocrinol..

[24] Tkachuk V.A., Vorotnikov A.V. (2014). Diabetes mellitus..

[25] Hirabara S.M., Gorjão R., Vinolo M.A., Rodrigues A.C., Nachbar R.T., Curi R. (2012). J. Biomed. Biotechnol..

[26] Rosen E.D., Spiegelman B.M. (2014). Cell..

[27] Luo L., Liu M. (2016). J. Endocrinol..

[28] Friedman J.M., Halaas J.L. (1998). Nature.

[29] Giralt M., Cereijo R., Villarroya F. (2016). Handb. Exp. Pharmacol..

[30] Scherer P.E. (2006). Diabetes..

[31] Stern J.H., Rutkowski J.M., Scherer P.E. (2016). Cell Metab..

[32] Flenkenthaler F., Ländström E., Shashikadze B., Backman M., Blutke A., Philippou-Massier J., Renner S., Hrabe de Angelis M., Wanke R. (2021). Front. Med..

[33] Rajala M.W., Lin Y., Ranalletta M., Yang X.M., Qian H., Gingerich R., Barzilai N., Scherer F.E. (2002). Mol. Endocrinol..

[34] Kharitonenkov A., Shiyanova T.L., Koester A., Ford A.M., Micanovic R., Galbreath E.J., Sandusky G.E., Hammond L.J., Moyers J.S., Owens R.A. (2005). J. Clin. Invest..

[35] Zhang Y., Proenca R., Maffei M., Barone M., Leopold L., Friedman J.M. (1994). Nature.

[36] Schaab M., Kratzsch J. (2015). Best Pract. Res. Clin. Endocrinol. Metab..

[37] Trusov N.V., Apryatin S.A., Gorbachev A.Yu., Naumov V.A., Mzhelskaya K.V., Gmoshinski I.V. (2018). Problems Endocrinol..

[38] Scherer P.E. (2016). Diabetes..

[39] Chawla A., Nguyen K.D., Goh Y.P. (2011). Nat. Rev. Immunol..

[40] Hotamisligil G.S., Shargill N.S., Spiegelman B.M. (1993). Science..

[41] Cai Z., Huang Y., He B. (2022). Cells..

[42] Turer A.T., Khera A., Ayers C.R., Turer C.B., Grundy S.M., Vega G.L., Scherer P.E. (2011). Diabetologia..

[43] Ahl S., Guenther M., Zhao Sh., James R., Marks J., Szabo A., Kidambi S. (2015). J. Clin. Endocrinol. Metab..

[44] Rajala M.W., Lin Y., Ranalletta M., Yang X.M., Qian H., Gingerich R., Barzilai N., Philipp E., Scherer Ph.E. (2002). Mol. Endocrinol..

[45] Steppan C.M., Bailey S.T., Bhat S., Brown E.J., Banerjee R.R., Wright C.M., Patel H.R., Ahima R.S., Lazar M.A. (2001). Nature.

[46] Aguilar-Salinas C.A., García E., Robles L., Riaño D., Ruiz-Gomez D.G., García-Ulloa A.C., Melgarejo M.A., Zamora M., Guillen-Pineda L., Mehta R. (2008). J. Clin. Endocrinol. Metab..

[47] Stefan N. (2020). Lancet Diabetes Endocrinol..

[48] Schleinitz D., Krause K., Wohland T., Gebhardt C., Linder N., Stumvoll M., Blüher M., Bechmann I., Kovacs P., Gericke M., Tönjes A. (2020). Eur. J. Hum. Genet..

[49] Raajendiran A., Krisp C., De Souza D.P., Ooi G., Burton P., Taylor R.A., Molloy M.P., M.J. I.O. (2021). Am. J. Physiol. Endocrinol. Metab..

[50] Gastaldelli A., Gaggini M., DeFronzo R. (2017). Curr. Opin. Clin. Nutr. Metab. Care..

[51] Guilherme A., Henriques F., Bedard A.H., Czech M.P. (2019). Nat. Rev. Endocrinol..

[52] Duvnjak L., Duvnjak M. (2009). J. Physiol. Pharmacol..

[53] Gibbs R.A., Weinstock G.M., Metzker M.L., Muzny D.M., Sodergren E.J., Scherer S., Scott G., Steffen D., Worley K.C., Burch P.E. (2004). Nature.

[54] Lopez-Jaramillo P., Gomez-Arbelaez D., Lopez-Lopez J., López-López C., Martínez-Ortega J., Gómez-Rodríguez A., Triana-Cubillos S. (2014). Horm. Mol. Biol. Clin. Investig..

[55] Perez-Perez A., Vilarino-Garcia T., Fernandez-Riejos P., Martín-González J., Segura-Egea J.J., Sánchez-Margalet V. (2017). Cytokine Growth Factor Rev..

[56] Yoshida S., Tanaka H., Oshima H., Yamazaki T., Yonetoku Y., Ohishi T. (2010). Biochem. Biophys. Res. Commun..

[57] Gault V.A., Kerr B.D., Harriott P., Flatt P.R. (2011). Clin. Sci. (London)..

[58] Park J.S., Rhee S.D., Kang N.S., Jung W.H., Kim H.Y., Kim J.H. (2011). Biochem. Pharmacol..

[59] Zhang Y., Proenca R., Maffei M., Barone M., Leopold L., Friedman J.M. (1994). Nature.

[60] Lindstrom P. (2007). Scientificworld Journal..

[61] Fang J.Y., Lin C.H., Huang T.H., Chuang S.Y. (2019). Nutrients..

[62] Chen H., Charlat O., Tartaglia L.A., Woolf E.A., Weng X., Ellis S.J., Lakey N.D., Culpepper J., Moore K.J., Breitbart R.E. (1996). Cell..

[63] King A.J.F. (2012). Br. J. Pharmacol..

[64] Phillips M.S., Liu Q., Hammond H.A., Dugan V., Hey P.J., Caskey C.J., Hess J.F. (1996). Nat. Genet..

[65] Pick A., Clark J., Kubstrup C., Levisetti M., Pugh W., Bonner-Weir S., Polonsky K.S. (1998). Diabetes..

[66] Shibata T., Takeuchi S., Yokota S., Kakimoto K., Yonemori F., Wakitani K. (2000). Br. J. Pharmacol..

[67] Leiter E.H. (2009). Methods Mol. Biol..

[68] Chakraborty G., Thumpayil S., Lafontant D.E., Woubneh W., Toney J.H. (2009). Lab. Anim. (N.Y.)..

[69] Kawano K., Hirashima T., Mori S., Natori T. (1994). Diabetes. Res. Clin. Pract..

[70] Clee S.M., Attie A.D. (2007). Endocr. Rev..

[71] Chen W., Zhou X.B., Liu H.Y., Xu C., Wang L.L., Li S. (2009). Br. J. Pharmacol..

[72] Fukaya N., Mochizuki K., Tanaka Y., Kumazawa T., Jiuxin Z., Fuchigami M., Toshinao Goda T. (2009). Eur. J. Pharmacol..

[73] Mochizuki K., Fukaya N., Tanaka Y., Fuchigami M., Goda T. (2011). Metabolism..

[74] Guo K., Yu Y.H., Hou J., Zhang Y. (2010). Nutr. Metab. (London)..

[75] Jia D., Yamamoto M., Otani M., Otsuki M. (2004). Metabolism..

[76] Ishiyama S., Kimura M., Nakagawa T., Fujimoto Y., Uchimura K., Kishigami S., Mochizuki K. (2021). Front. Endocrinol. (Lausanne)..

[77] Kottaisamy C.P.D., Raj D.S., Prasanth Kumar V., Sankaran U. (2021). Lab. Anim. Res..

[78] Loza-Rodríguez H., Estrada-Soto S., Alarcón-Aguilar F.J., Huang F., Aquino-Jarquín G., Fortis-Barrera Á., Giacoman-Martínez A., Almanza-Pérez J.C. (2020). Eur. J. Pharmacol..

[79] Yoshinari O., Igarashi K. (2011). Br. J. Nutr..

[80] Xu T.Y., Chen R.H., Wang P., Zhang R.Y., Ke S.F., Miao C.Y. (2010). Clin. Exp. Pharmacol. Physiol..

[81] Itoh T., Kobayashi M., Horio F., Furuichi Y. (2009). Nutrition..

[82] Kluth O., Mirhashemi F., Scherneck S., Kaiser D., Kluge R., Neschen S., Joost H.G., Schürmann A. (2011). Diabetologia..

[83] Lee M.Y., Shim M.S., Kim B.H., Hong S.W., Choi R., Lee E.Y., Nam S.M., Kim G.W., Shin J.Y., Shin Y.G. (2011). Diabetes. Metab. J..

[84] Choi R., Kim B.H., Naowaboot J., Lee M.Y., Hyun M.R., Cho E.J., Lee E.S., Lee E.Y., Yang Y.C., Chung C.H. (2011). Exp. Mol. Med..

[85] Fang R.C., Kryger Z.B., Buck D.W., De la Garza M., Galiano R.D., Mustoe T.A. (2010). Wound. Repair. Regen..

[86] Rai V., Moellmer R., Agrawal D.K. (2022). Mol. Cell. Biochem..

[87] Surwit R.S., Kuhn C.M., Cochrane C., McCubbin J.A., Feinglos M.N. (1988). Diabetes..

[88] Winzell M.S., Ahren B. (2004). Diabetes..

[89] Surwit R.S., Feinglos M.N., Rodin J., Sutherland A., Petro A.E., Opara E.C., Kuhn C.M., Rebuffé-Scrive M. (1995). Metabolism..

[90] Bachmanov A.A., Reed D.R., Tordoff M.G., Price R.A., Beauchamp G.K. (2001). Physiol. Behav..

[91] Sclafani A. (2007). Physiol. Behav..

[92] Almind K., Kahn C.R. (2004). Diabetes..

[93] Torre-Villalvazo I., Cervantes-Pérez L.G., Noriega L.G., Jiménez J.V., Uribe N., Chávez-Canales M., Tovar-Palacio C., Marfil-Garza B.A., Torres N., Bobadilla N.A. (2018). Am. J. Physiol. Endocrinol. Metab..

[94] Lackey D.E., Lazaro R.G., Li P., Johnson A., Hernandez-Carretero A., Weber N., Vorobyova I., Tsukomoto H., Osborn O. (2016). Am. J. Physiol. Endocrinol. Metab..

[95] Pereira-Silva D.C., Machado-Silva R.P., Castro-Pinheiro C., Fernandes-Santos C. (2019). Int. J. Exp. Pathol..

[96] Burcelin R., Crivelli V., Dacosta A., Roy-Tirelli A., Thorens B. (2002). Am. J. Physiol. Endocrinol. Metab..

[97] Noda K., Melhorn M.I., Zandi S., Frimmel S., Tayyari F., Hisatomi T., Almulki L., Pronczuk A., Hayes K.C., Hafezi-Moghadam A. (2010). FASEB J..

[98] Noda K., Nakao S., Zandi S., Sun D., Hayes K.C., Hafezi-Moghadam A. (2014). FASEB J..

[99] Sinasac D.S., Riordan J.D., Spiezio S.H., Yandell B.S., Croniger C.M., Nadeau J.H. (2016). Int. J. Obes. (London)..

[100] Pirmardan R.E., Barakat A., Zhang Y., Naseri M., Hafezi-Moghadam A. (2021). FASEB J..

[101] Chaabo F., Pronczuk A., Maslova E., Hayes K. (2010). Nutr. Metab. (London)..

[102] Weir G.C., Marselli L., Marchetti P., Katsuta H., Jung M.H., Bonner-Weir S. (2009). Diabetes. Obes. Metab..

[103] Goto Y., Kakizaki M., Masaki N. (1976). Tohoku J. Exp. Med..

[104] Portha B., Giroix M.H., Serradas P., Gangnerau M.N., Movassat J., Rajas F., Bailbe D., Plachot C., Mithieux G., Marie J.C. (2001). Diabetes..

[105] Ostenson C.G., Efendic S. (2007). Diabetes. Obes. Metab..

[106] Portha B., Lacraz G., Kergoat M., Homo-Delarche F., Giroix M.H., Bailbe D., Gangnerau M.N., Dolz M., Tourrel-Cuzin C., Movassat J. (2009). Mol. Cell. Endocrinol..

[107] Kottaisamy C.P.D., Raj D.S., Kumar P.V., Sankaran U. (2021). Lab. Anim. Res..

[108] Zhao J.D., Li Y., Sun M., Yu C.J., Li J.Y., Wang S.H., Yang D., Guo C.L., Du X., Zhang W.J. (2021). World. J. Gastroenterol..

[109] Szkudelska K., Deniziak M., Sassek M., Szkudelski I., Noskowiak W., Szkudelski T. (2021). Int J. Mol. Sci..

[110] Ehses J.A., Lacraz G., Giroix M.H., Schmidlin F., Coulaud J., Kassis N., Irminger J.C., Kergoat M., Portha B., Homo-Delarche F. (2009). Proc. Natl. Acad. Sci. USA..

[111] Okada S., Saito M., Kinoshita Y., Satoh I., Kawaba Y., Hayashi A., Oite T., Satoh K., Kanzaki S. (2010). Biomed. Res..

[112] Burillo J., Marqués P., Jiménez B., González-Blanco C., Benito M., Guillén C. (2021). Cells..

[113] Asiri M.M.H., Engelsman S., Eijkelkamp N., Höppener J.W.M. (2020). Cells..

[114] Hoppener J.W., Oosterwijk C., van Hulst K.L., Verbeek J.S., Capel P.J., de Koning E.J., Clark A., Jansz H.S., Lips C.J. (1994). J. Cell. Biochem..

[115] Zhang X.X., Pan Y.H., Huang Y.M., Zhao H.L. (2016). World. J. Diabetes..

[116] Matveyenko A.V., Butler P.C. (2006). ILAR. J..

[117] Matveyenko A.V., Gurlo T., Daval M., Butler A.E., Butler P.C. (2009). Diabetes..

[118] Hara M., Wang X., Kawamura T., Bindokas V.P., Dizon R.F., Alcoser S.Y., Magnuson M.A., Bell G.I. (2003). Am. J. Physiol. Endocrinol. Metab..

[119] Sanavia T., Huang C., Manduchi E., Xu Y., Dadi P.K., Potter L.A., Jacobson D.A., Di Camillo B., Magnuson M.A., Stoeckert C.J. Jr., Gu G. (2021). Front. Cell. Dev. Biol..

[120] Sasaki S., Lee M.Y.Y., Wakabayashi Y., Suzuki L., Winata H., Himuro M., Matsuoka T.A., Shimomura I., Watada H., Lynn F.C. (2022). Diabetologia..

[121] Habener J.F., Kemp D.M., Thomas M.K. (2005). Endocrinology..

[122] Oliver-Krasinski J.M., Kasner M.T., Yang J., Crutchlow M.F., Rustgi A.K., Kaestner K.H., Stoffers D.A. (2009). J. Clin. Invest..

[123] Wang Q., Jin T. (2009). Islets..

[124] Wang B., Chandrasekera P.C., Pippin J.J. (2014). Curr. Diabetes. Rev..

[125] Masiello P., Broca C., Gross R., Roye M., Manteghetti M., Hillaire-Buys D., Novelli M., Ribes G. (1998). Diabetes..

[126] Junod A., Lambert A.E., Stauffacher W., Renold A.E. (1969). J. Clin. Invest..

[127] Schein P.S., Cooney D.A., Vernon M.L. (1967). Cancer Research.

[128] Ghasemi A., Khalifi S., Jedi S.S. (2014). Acta. Physiologica Hungarica..

[129] Masiello P. (2006). Int. J. Biochem. Cell. Biol..

[130] Reed M.J., Meszaros K., Entes L.J., Claypool M.D., Pinkett J.G., Gadbois T.M., Reaven G.M. (2000). Metabolism..

[131] Chao P.C., Li Y., Chang C.H., Shieh J.P., Cheng J.T., Cheng K. C. (2018). Biomed. Pharm..

[132] Gheibi S., Kashfi K., Ghasemi A. (2017). Biomed. Pharm..

[133] Furman B.L. (2021). Curr. Protoc..

[134] Zhang M., Lv X.Y., Li J., Xu Z.G., Chen L. (2008). Exp. Diab. Res..

[135] Yorek M.A. (2016). Int. Rev. Neurobiol..

[136] Elsner M., Tiedge M., Guldbakke B., Munday R., Lenzen S. (2002). Diabet..

[137] Lenzen S. (2008). Diabetologia..

[138] Olefsky J., Crapo P.A., Ginsberg H., Reaven G.M. (1975). Metabolism..

[139] Kibenge M.T., Chan C.B. (2002). Metabolism..

[140] Srinivasan K., Viswanad B., Asrat L., Kaul C., Ramarao P. (2005). Pharm. Res..

[141] Premilovac D., Gasperini R.J., Sawyer S., West A., Keske M.A., Taylor B.V., Foa L. (2017). Sci. Rep..

[142] Skovsø S. (2014). J. Diab. Inv..

[143] de la Garza-Rodea A.S., Knaän-Shanzer S., den Hartigh J.D., Verhaegen A.P., van Bekkum D.W. (2010). J. Am. As. Lab. Anim. Science..

[144] Furman B.L., Candasamy M., Bhattamisra S.K., Veettil S.K. (2020). J. Ethnopharmacol..

[145] Wu K.K., Huan Y. (2007). Atherosclerosis..

[146] Heydemann A. (2016). J. Diabetes Res..

[147] Islam M.S., du Loots T. (2009). Meth. Find. Exp. Clin. Pharmacol..

[148] Iannaccone P.M., Jacob H.J. (2009). Dis. Model Mech..

[149] Sharma P., Garg A., Garg S., Singh V. (2016). Asian J. Biomat. Res..

[150] Bryda E.C. (2013). Mol. Med..

[151] Franconi F., Seghieri G., Canu S., Straface E., Campesi I., Malorni W. (2008). Pharm. Res..

[152] Arai I., Miyazaki N., Seino Y., Fukatsu A. (2007). Biosci. Biotech. Biochem..

[153] Mestas J., Hughes C.C. (2004). J. Immunol..

